# Synthesis of pH-Sensitive and Self-Fluorescent Polymeric Micelles Derived From Rosin and Vegetable Oils *via* ATRP

**DOI:** 10.3389/fbioe.2021.753808

**Published:** 2021-11-02

**Authors:** Juan Yu, Chaoqun Xu, Chuanwei Lu, Qian Liu, Jifu Wang, Fuxiang Chu

**Affiliations:** ^1^ Key Laboratory of Forestry Genetics and Biotechnology, Jiangsu Province Key Laboratory of Green Biomass-based Fuels and Chemicals, Ministry of Education, Jiangsu Co-Innovation Center of Efficient Processing and Utilization of Forest Resources, College of Chemical Engineering, Nanjing Forestry University, Nanjing, China; ^2^ National Engineering Laboratory of Biomass Chemical Utilization, Key and Laboratory of Forest Chemical Engineering, SFA, Key Laboratory of Biomass Energy and Material, Institute of Chemical Industry of Forest Products, CAF, Nanjing, China; ^3^ Research Center for Nanophotonic and Nanoelectronic Materials, School of Materials Science and Engineering, Suzhou University of Science and Technology, Suzhou, China

**Keywords:** pH-sensitive, self-fluorescence, polymeric micelles, rosin, vegetable oils, ATRP, doxorubicin

## Abstract

Preparation and application of sustainable polymers derived from renewable resources are of great significance. The aim of this study is to synthesize a kind of sustainable polymeric micelles from rosin and vegetable oils *via* atom transfer radical polymerization (ATRP) and to investigate the doxorubicin delivery properties of these micelles. Dehydroabietic acid–based poly lauryl methacrylate (DA-PLMA) with narrow PDI of 1.13 was prepared in a well-controlled process using rosin as an ATRP initiator. Thereafter, carboxylic groups were introduced to form poly methacrylic acid (PMAA) moieties in DA-PLMA polymer *via* acid hydrolysis. The resulted DA-PLMA-PMAA could self-assemble in water to form pH-dependent polymeric micelles with a diameter of ∼65 nm and PDI as low as 0.105. Owing to the existence of rosin, DA-PLMA-PMAA micelles also showed self-fluorescence properties. In addition, Dox-loaded micelles were prepared in aqueous solution with the drug-loading capacity as high as 16.0% and showed sustained-release characteristics. These results demonstrate great promise for designing polymeric micellar from rosin and vegetable oils.

## Introduction

The application of renewable raw materials (included natural polymers and chemicals) can take advantage of the synthetic potential of nature and avoid or minimize CO_2_ emission, which is of great significance for carbon emissions and carbon neutralization (Meier et al., 2007; Thyavihalli Girijappa et al., 2019). Renewable natural polymers such as cellulose, hemicellulose, starch, chitin, lignin, and natural rubber have been widely explored in chemical industry for the partial replacement of fossil fuel–based polymeric materials (Biermann et al., 2011; Zhang et al., 2017; Wang J. et al., 2020; Sun et al., 2020). However, natural rosin, a kind of important natural chemicals, which can be easily obtained from pine resins and some other related plants, has been modified to be various kinds of fine chemicals and monomers to replace petrochemicals in industry (Cai et al., 2021). These rosin-based monomers could be polymerized *via* controlled radical polymerization resulting in a class of well-defined rosin-derived polymers. Owing to the good thermal properties, excellent hydrophobicity, biocompatibility, and UV absorption properties, these renewable rosin-derived polymers could be potential candidates for thermoplastic elastomers. Recently, rosin was transferred to an atom transfer radical polymerization (ATRP) initiator which provides an alternative avenue for the designation of rosin-based polymers (with rosin content less than 10 wt%) to achieve the improvement in thermal, UV-blocking, and mechanical performance (Yu et al., 2021).

Polymeric micelles formed by self-assembly of amphiphilic block copolymers have been widely applied as excellent carriers for hydrophobic drugs because they have high drug-loading capacity, sustained release manner (owing to their hydrophobic core), and the stabilization ability in aqueous solution (owing to their hydrophilic shell) (Tyrrell et al., 2010; Tang et al., 2011; Bastakoti et al., 2013; Sun et al., 2018). Therefore, a number of amphiphilic block copolymers have been synthesized and reported to form a hydrophilic shell of micelles in aqueous solution. Regarding amphiphilic block copolymers for polymeric micelles, PEG is the most widely used hydrophilic block; the commonly reported hydrophobic blocks are poly (propylene oxide) (PPO), poly (ε-caprolactone) (PCL) and poly (lactide) (PLA), and poly lauryl methacrylate (PLMA) (Tyrrell et al., 2010; Tang et al., 2011; Hattori et al., 2017). There are various method choices for the synthesis of amphiphilic block copolymers, including free radical polymerization (Debele et al., 2017), ring-opening polymerization (Dai et al., 2011), reversible addition–fragmentation chain transfer (RAFT) (Wang C. et al., 2020), and ATRP (Oh et al., 2007).

However, most of the reported polymeric micelles were prepared from fossil fuel–based polymers. Research works focusing on polymeric micelles from renewable resource were rarely reported. Hespel et al. (2012) reported the synthesis of pH-sensitive micelles from renewable linseed oil *via* ATRP. The amphiphilic copolymers from linseed oils were prepared by ATRP of tert-butyl acrylate (tBA) from a linseed oil initiator and subsequent acidolysis of the PtBA block into poly(acrylic acid) (PAA). Compared to amphiphilic block copolymers, the preparation of amphiphilic random copolymers is much easier. In addition, it is reported that amphiphilic copolymers obtained by copolymerizing a hydrophilic poly(ethylene glycol) (PEG) monomer and a hydrophobic lauryl monomer with a random monomer sequence could produce uniform micelles in water, and the micelle’s size was determined by the composition (Hattori et al., 2017). These results promote the fabrication of polymeric micelles derived from renewable vegetable oil resources.

**SCHEME 1:**
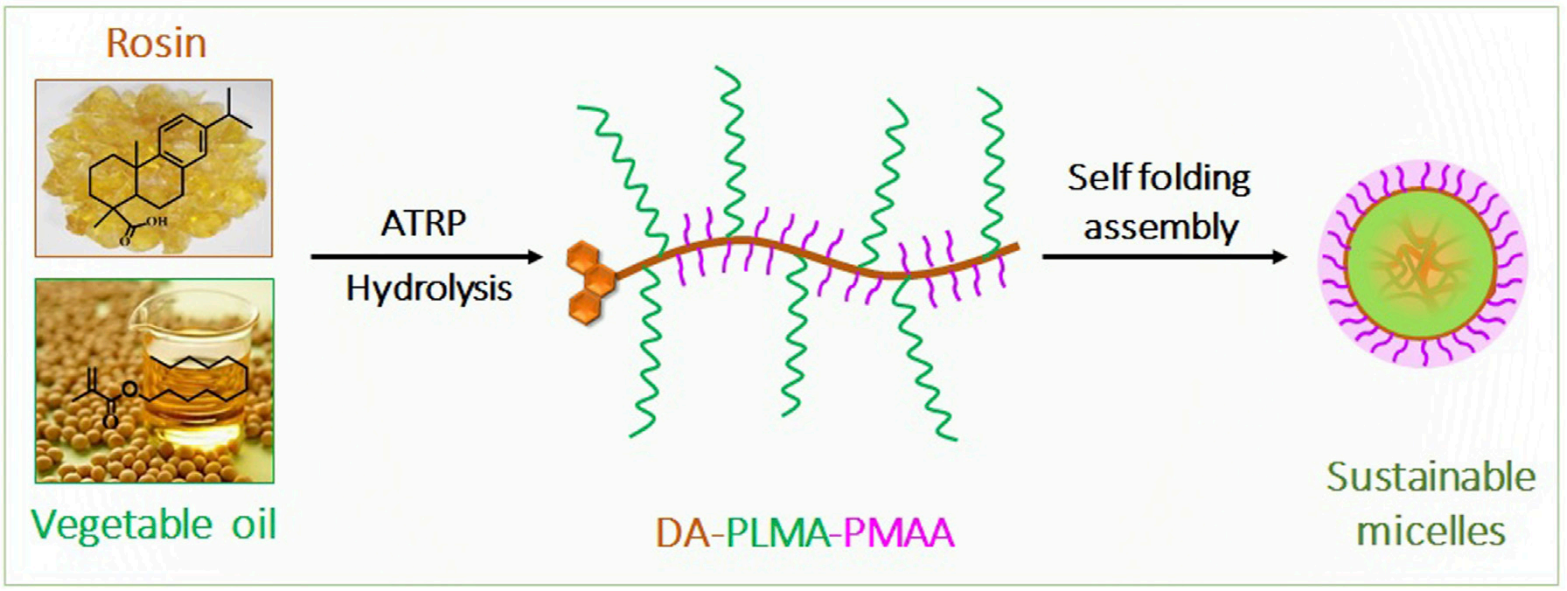
Schematic illustration of synthesis of DA-PLMA-PMAA sustainable micelles from rosin and vegetable oil.

The objective of this study was to develop polymeric micelles based on rosin and vegetable oils as a drug carrier and to reveal the role of rosin in polymeric micelles. As shown in Scheme 1, a rosin-based initiator (2-BriBEDA) and lauryl methacrylate (LMA, derived from vegetable oil) were used as raw materials. Hydrophobic dehydroabietic acid–based poly (lauryl methacrylate) (DA-PLMA) was prepared *via* ATRP using 2-BriBEDA as an initiator. After acid hydrolysis, poly methacrylic acid (PMAA) moieties were introduced in PLMA blocks, resulting in an amphiphilic random copolymer (DA-PLMA-PMAA). The self-assembly of DA-PLMA-PMAA into polymeric micelles was verified and characterized. Hydrophobic drug doxorubicin (Dox) was encapsulated into the resulted micelles. This novel type of biomass-based polymeric micelles exhibit pH-sensitivity, self-fluorescent, high drug-loading level capacity, and aqueous stability, making it an extremely promising nanoplatform for the drug carrier field.

## Experimental

### Materials

A rosin-based ATRP initiator (2-BriBEDA) was prepared using dehydroabietic acid (DA, main content of rosin) as a raw material according to our previous study (Yu et al., 2021). *N,N,N′,N″,N″*-pentamethyldiethylenetriamine (PMDETA, 99%, Aladdin Industrial Inc.), lauryl methacrylate (LMA, 96%, Aldrich), and CuBr (99.999%, Aldrich) were used as received. Doxorubicin hydrochloride (Dox HCl) was purchased from Beijing Huafeng United Technology Company. Tetrahydrofuran (THF, AR reagents), dichloromethane (DCM, AR reagents), methanol (AR reagents), and petroleum ether (AR reagents) were purchased from Nanjing Reagent Chemical Co. Ltd. Anisole was dried over 4A molecular sieves and then distilled before use.

### Characterization

An FT-IR analysis of rosin-based polymers before and after acid hydrolysis was performed using a Nicolet iS10 FT-IR spectrometer by an attenuated total reflectance method.

A ^1^H NMR analysis of rosin-based polymers before and after acid hydrolysis was carried out on a Bruker DMX 300 NMR spectrometer, and CDC1_3_ or DMSO-d_6_ was used as the solvent.

Gel permeation chromatography (GPC) was performed at 40°C to measure molecular weight and molecular weight distribution of rosin-based polymers. All samples were filtered over a microfilter with a pore size of 0.22 µm (Nylon, Millex-HN 13 mm Syringes Filters, Millipore). A Malvern Viscotek 3580 System, a Viscotek GPC2502 refractive detector, and a GPC1007 pump were involved. An HPLC-grade THF was used as the eluent, and the flow rate was 1 ml/min. Monodispersed polystyrene (PS) was used as the standard to generate the calibration curve.

A UV-visible spectrophotometer (UV/vis) analysis was performed by recording the solution of rosin-based polymers before and after acid hydrolysis in THF on a Shimadzu UV-1800, Shimadzu Scientific Instruments Incorporated in a double-beam mode, whereas pure THF was used as a reference of the copolymer solution while the wavelength is between 200 and 400 nm.

Fluorescence (FL) spectra were used to characterize the fluorescence properties, which were measured on a HITACHI F-4500 spectrofluorometer with the band widths of 10 nm for excitation and 2.5 nm for emission, where the excitation wavelength (lex) was 360 nm. The samples were dissolved in THF with a concentration of 2 mg/ml.

Surface tension of the micelle’s solution was measured by using the Wilhelmy plate technique at 25°C. The tensiometer was calibrated against water before measurements. The platinum plate was always cleaned and heated to red color with an alcohol lamp.

Transmission electron microscope (TEM) images were obtained by a JEOL JEM-2011 instrument operated at 100 kV. For TEM studies, a drop of micellar solution was deposited on an electron microscopy copper grid coated with a carbon film, and the water was evaporated at room temperature.

The sizes, size distributions, and zeta-potentials of prepared micelles were determined using a Zetasizer Nano-ZS Instrument (ZEM4228, Malvern Instruments, United Kingdom). Each sample was equilibrated at 25°C for 1 min before measurement, and five sets of time-averaged measurements were taken. A 633 nm He–Ne laser was the light source.

### Synthesis of DA-PLMA *via* ATRP

**FIGURE 1:**
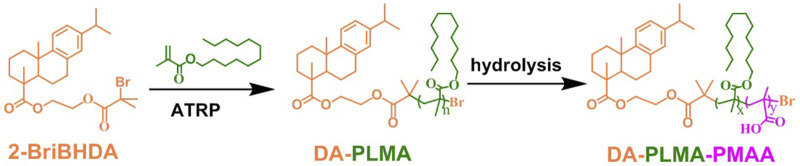
Schematic illustration of synthesis of DA-PLMA from DA-based initiators, and partial hydrolysis of DA-PLMA to prepare DA-PLMA-PMAA.

2-BriBHDA was used as the initiator to initiate the polymerization of vegetable oil–based monomer lauryl methacrylate (LMA) *via* ATRP. As shown in Figure 1, for a typical polymerization procedure, a mixture of LMA (2.57 g, 1.01 × 10^−2^ mol), PMDETA (9.13 mg, 5.07 × 10^−5^ mol), 2-BriBHDA (25 mg, 5.07 × 10^−5^ mol), and anisole (3 ml) was charged into a round-bottom flask and degassed by three freeze–pump–thaw cycles. Thereafter, the mixture was transferred into a Schlenk flask that contained Cu(I)Br (7.5 mg, 5.07 × 10^−5^ mol) under nitrogen and soon placed in an oil bath set at 100°C. Periodically, samples were withdrawn from the Schlenk flask *via* a syringe under nitrogen to determine the monomer conversion, molecular weight, and polydispersity of the resulted polymer. The polymerization was stopped by pouring the reaction mixture with THF. After passing through an alkaline alumina column, the product was precipitated into methanol, collected, and dried under vacuum until constant weight.

### Preparation of DA-PLMA-PMAA by Acid Hydrolysis of DA-PLMA

1.5 ml of concentrated sulfuric acid was added to a Teflon centrifugal tube containing 100 mg DA-PLMA copolymer; subsequently, the reaction mixture was stirred in 45°C water bath for 12 h. DA-PLMA-PMAA were obtained by filtration and washed with a plenty of water/methanol mixture prior to being lyophilized (Semen, 1969).

### Micelle Formation

Briefly, the DA-PLMA-PMAA copolymer (10 mg) was dissolved in 2 ml THF to give 5 mg/ml solutions, followed by the dropwise addition into distilled water with vigorous stirring until the concentration reached to 0.1 mg/ml. The mixture was left to stand at room temperature for 24 h and was filtered over a microfilter with a pore size of 0.45 µm prior to the subsequent measurement. The polymeric micelle with concentration of 1 mg/ml was prepared in a similar way.

### Preparation of Dox-Loaded Micelles and *In Vitro* Drug Release

Dox-loaded micelles were prepared by an incubation method according to our previous study (Nan et al., 2014). The micelle solution (prepared above) was mixed with Dox solution of various predetermined concentrations at room temperature. Subsequently, in order to allow the DOX/micelle mixture to reach an equilibrium state, the mixed solution was incubated at 37°C for 12 h. To determine the entrapment efficiency (EE) and drug-loading efficiency (LE), the drug-loaded micelle solution was centrifuged, and the amount of free Dox in the solution was analyzed by using a UV-vis spectrometer at 495 nm, using a standard calibration curve experimentally obtained with DOX/water solution. The drug-loading capacity (LC) and drug-loading efficiency (LE) were calculated according to the following formulas:

LE(%)=Weight of DOX Loaded in micelles/Weight of feeding DOX∗100%, (1)

LC(%)=Weight of DOX Loaded in micelles/Weight of micelles∗100%. (2)

The centrifuged DOX/micelle mixture was dispersed in water and then loaded into a dialysis bag (molecular weight cut-off: 14 kDa) and dialyzed against PBS (pH = 7.4) in a beaker at 37°C for *in vitro* drug release. At selective time intervals, 3 ml of solution was withdrawn from the release medium and replaced with 3 ml fresh PBS. The DOX content in the samples was analyzed using the UV-vis spectrophotometer at 495 nm.

## Results and Discussion

### Synthesis of DA-PLMA *via* ATRP

**FIGURE 2:**
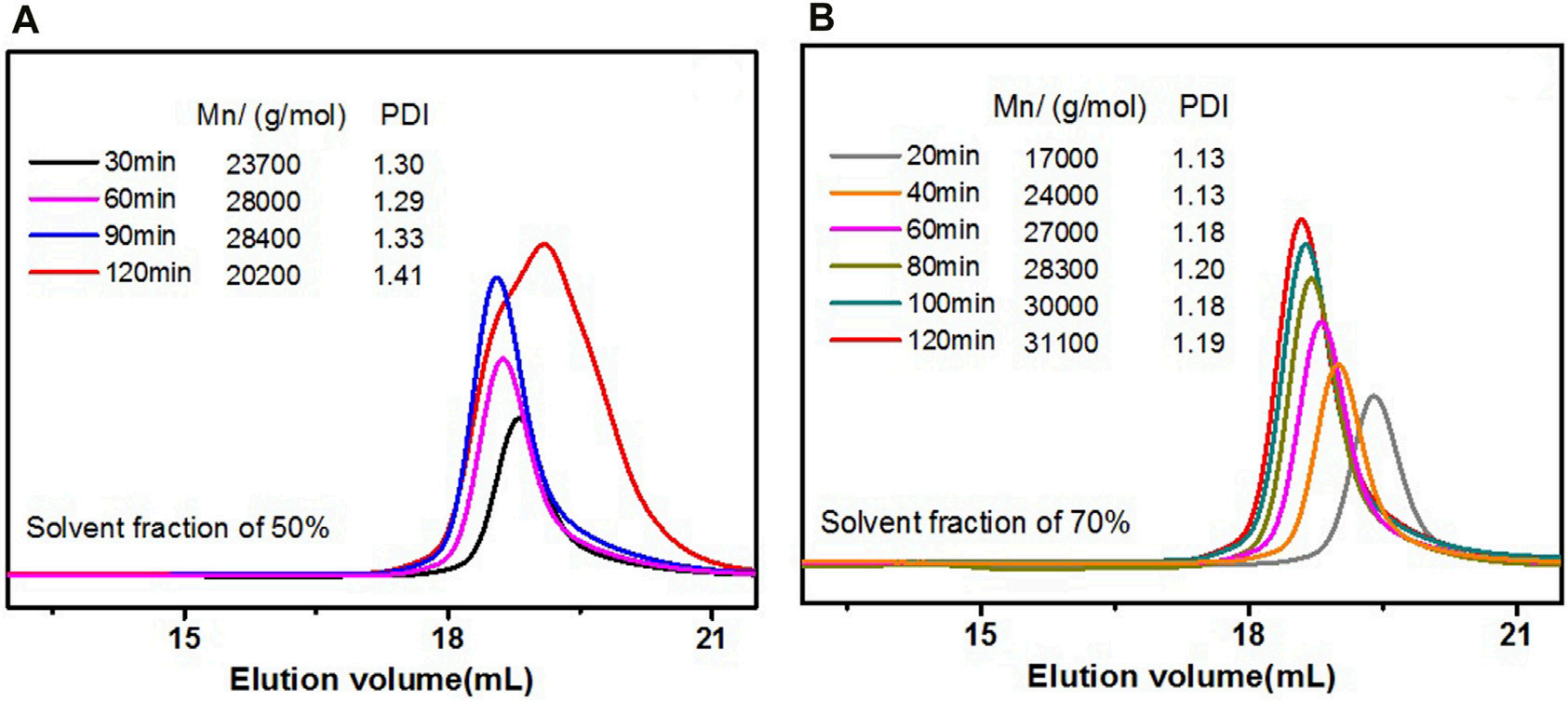
GPC traces of DA-PLMA polymers and Mn/PDI using anisole as a solvent with different reaction times: **(A)** solvent fraction of 50% and **(B)** solvent fraction of 70%.

Given that the polydispersion index (PDI) of the polymer has a key effect on the PDI of polymeric micelles, the ATRP polymerization condition of DA-PLMA was optimized first to obtain narrow PDI for the polymers. As shown that PMDETA/CuBr is the best catalyst system for the rosin-based ATRP initiator (Yu et al., 2021), the influence of solvents was emphasized in this study. It is reported that for the polymerization of PLMA, the PDI of PLMA obtained in anisole was a little narrower than those obtained in the other investigated solvents (DMF, acetonitrile, benzene, or toluene) (Çayli and Meier, 2008). Therefore, anisole was chosen as the solvent in this condition. Two different solvent fractions were employed, respectively, with the same feeding ratio {[LMA]/[I]/[Cu(I)]/PMDETA = 200/1/1/1} as solvent fraction could affect ATRP equilibrium constants (K_ATRP_) (Wang et al., 2012). Commonly, the lower K_ATRP_ may lead to a better controlled polymerization. The results of Mn as well as PDI were monitored by GPC. As shown in Figure 2A, the GPC traces of DA-PLMA were symmetrical and monomodal peaks at first 60 min, but became wider after 60 min with a decrease of Mn and increase of PDI. The final products obtained at 120 min had a Mn of 20,200 g/mol and PDI of 1.41, which means that the ATRP polymerization of LMA was not controlled well in this condition. However, when the solvent fraction increased to 70%, all the GPC traces at different reaction times (from 20 to 120 min) showed symmetrical and monomodal peaks, Mn values changed linearly with the reaction time, and all the PDI values were not more than 1.2, confirming the controlled character of polymerization (Figure 2B). It should be noted that the reaction medium is initially a mixture of solvent/monomer, but it gradually changed to solvent/monomer/polymer as the polymerization proceeds. The polymer fraction increased with increasing reaction time, and so was the viscosity of the reaction medium, especially in the reaction with lower solvent fraction. The reason why higher solvent fraction leads to a better control of polymerization in this study is probably because the viscosity of the reaction medium was not increased so much that affects the polymerization. Thus, anisole (volume 70%) was used as the solvent in most cases.

**FIGURE 3:**
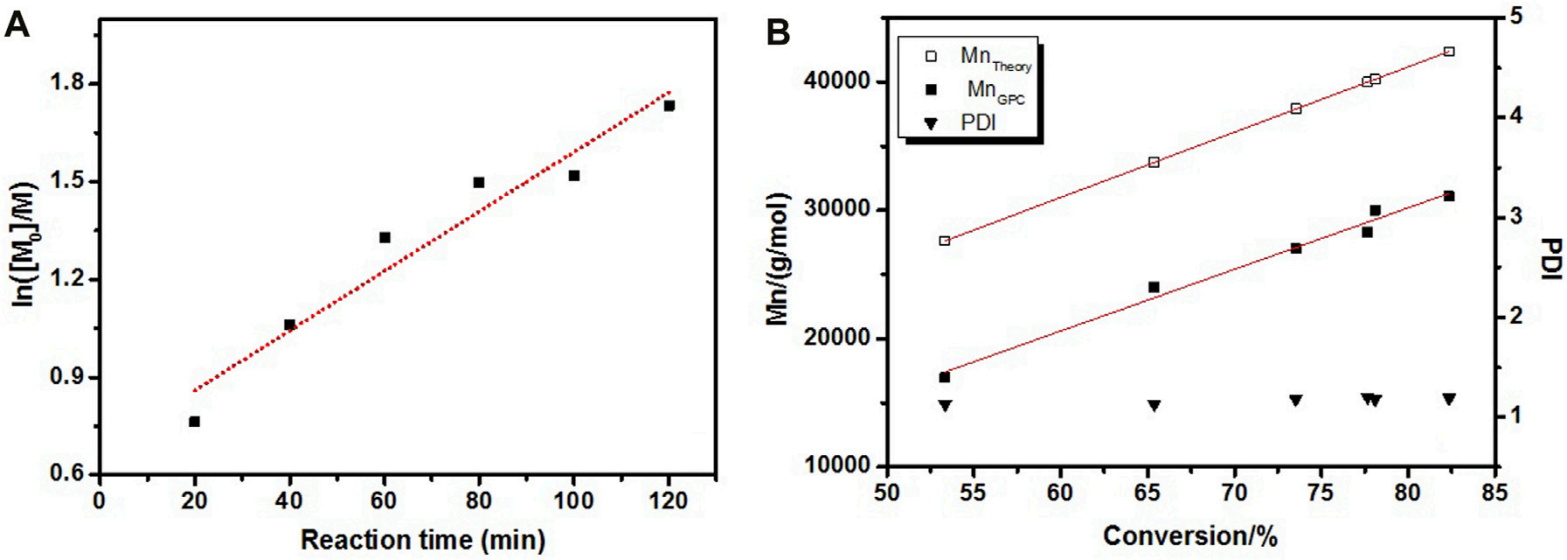
Kinetic plots for the polymerization of LMA **(A)** and evolution of the *M*_n_ (theoretical and GPC) and PDI **(B)**.

The controlled and living nature of polymerizations were verified through kinetic experiment which was monitored by analyzing the reaction mixture with ^1^H NMR spectroscopy and GPC. The monomer conversion was calculated *via* the ^1^H NMR spectrum of the reaction mixture according to Eq. 3 and GPC given Mn and PDI of resulted polymer. The semilogarithmic plot of monomer conversion vs. reaction time is shown in Figure 3A. The linear dependence of ln([M]_0_/[M]) on time suggested that the polymerization is a well-controlled process. The *M*_n_s obtained by GPC is shown in Figure 3B, and the molecular weight of copolymers increased with conversion. Meanwhile, the PDI remained less than 1.2 over the whole reaction, meaning a good control of the final copolymer composition (Hespel et al., 2012). In addition, the number average molecular weights of the copolymers determined by GPC were not in fair agreement with the theoretical values calculated by ^1^H NMR (Figure 3B). The lower molecular weights determined by GPC could be explained by a notion that molecular weights determined by GPC using PS standards correspond to PS-equivalent molecular weights (Netopilík and Kratochvíl, 2003). Therefore, it is the hydrodynamic volume disparity between PS and PLMA that leads to the discrepancy between theoretical and experimental *M*_*n*_s.

$$\text{LMA}\;\text{conversion}\left(\text{\%}\right)=\left(1-18/{\text{A}}_{1.31}*{\text{A}}_{5.54}\right)*100\%,$$ (3)

where A_1.31_ is the ^1^H NMR integration area (∼1.31 ppm) of methylene protons in the lauryl of LMA, A_5.54_ is the ^1^H NMR integration area (∼5.54 ppm) of unsaturated protons in LMA, and 18 is the number of protons of methylene protons in the lauryl of LMA.

### Chemical Structure of DA-PLMA and DA-PLMA-PMAA

Amphiphilic copolymers with both hydrophobic segments and hydrophilic segments could autonomously self-assemble to be micelles or vesicles in water *via* hydrophobic effects (Hattori et al., 2017). Therefore, DA-PLMA-PMAA was obtained by acid hydrolysis of the hydrophobic polymer DA-PLMA. During acid hydrolysis, hydrophobic lauryl was removed while the hydrophilic carboxyl group was introduced at the same time. ATR-FT-IR, ^1^H NMR, the UV/vis analysis, and GPC were employed to characterize the chemical structure of DA-PLMA before and after hydrolysis.

**FIGURE 4:**
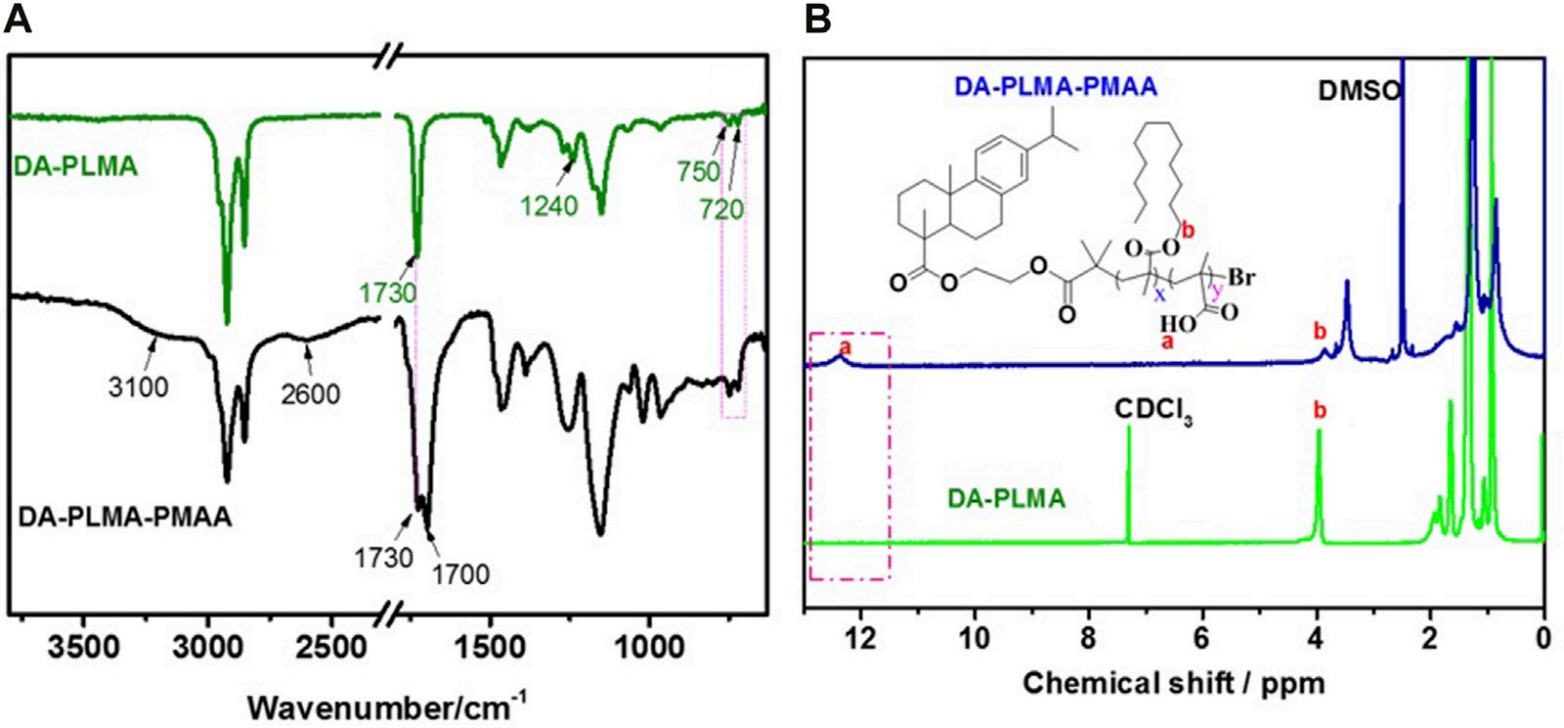
Spectrum of DA-PLMA before and after hydrolysis **(A)** FT-IR and **(B)** ^1^H NMR.

The ATR-FT-IR analysis (Figure 4A) of DA-PLMA showed the appearance of C=O signal peak at 1730 cm^−1^ as well as the peak of C-O-C at 1,240 cm^−1^ which ascribed to ester group in PLMA parts. The absorption peaks at around 720 cm^−1^ and 750 cm^−1^ correspond to the–CH_2_ group in the side lauryl chain of PLMA and the main chain of PLMA, respectively, while the appearance of absorption peaks at around 3,100 cm^−1^ (O-H), 2,600 cm^−1^ (O-H), and 1,700 cm^−1^ (C=O) in the spectra of hydrolysis polymer (DA-PLMA-PMAA) indicated the formation of carboxylic groups after hydrolysis; however, the peak at 1,730 cm^−1^ and 720 cm^−1^ still exist meant that the partial hydrolysis of DA-PLMA and the hydrophobic lauryl side chain was also retained in the resulted DA-PLMA-PMAA. The ^1^H NMR analysis (Figure 4B) showed the appearance of peak at 12.2 ppm corresponding to the protons of O-H moiety in carboxylic groups which verifies the successful introduction of carboxylic groups by acid hydrolysis. The –OCH_2_ protons of PLMA (3.9 ppm) and –CH_2_ protons (∼1.31 ppm) in lauryl side chains of LMA still present in the ^1^H NMR spectrum of DA-PLMA-PMAA, further confirming the partial hydrolysis of DA-PLMA. The absence of the protons of rosin (usually around 6.8–7.3 ppm) probably caused the entanglement of lauryl long side chains of PLMA around the rosin-based initiator, which could be seen by zooming in the ^1^H NMR spectrum (Yu et al., 2021).

**FIGURE 5:**
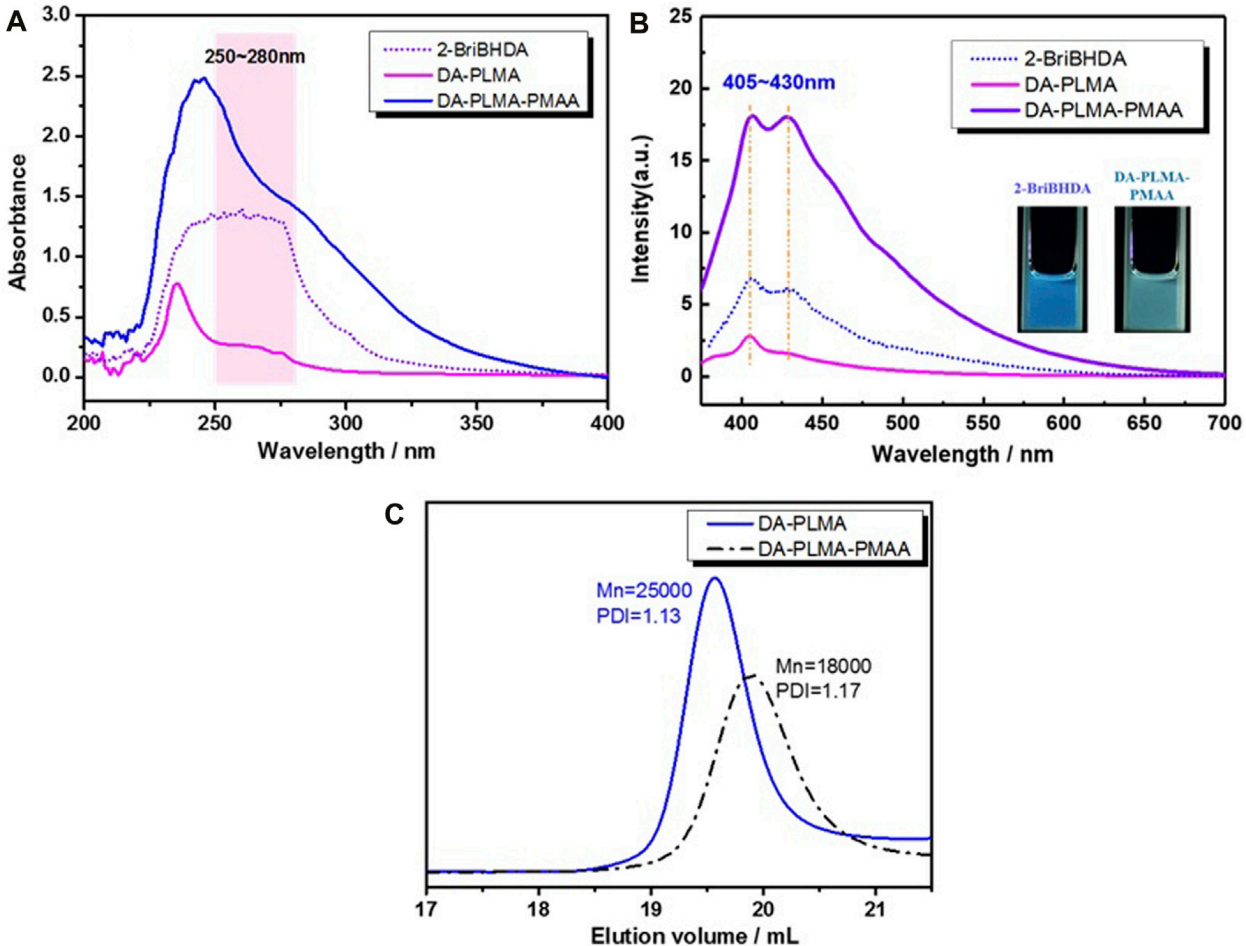
**(A)** UV absorption curves and **(B)** fluorescence spectra (inset: digital photograph taken under 365 nm) of the rosin-based initiator, DA-PLMA, and DA-PLMA-PMAA in THF; **(C)** GPC traces of DA-PLMA and DA-PLMA-PMAA.

As rosin and its derivatives have an absorption in the UV region (200–400 nm), the UV/vis analysis was further employed to confirm the existence of rosin in DA-PLMA and DA-PLMA-PMAA. As shown in Figure 5A, the rosin-based initiator (2-BriBHDA, 0.33 mg/ml) had a UV absorption peak around 250–280 nm. The obvious absorption around 250–280 nm was clearly presented in the UV spectrum of DA-PLMA (2 mg/ml). After acid hydrolysis, UV absorption intensity around 250–280 nm increased significantly for DA-PLMA-PMAA (2 mg/ml). This is caused by the fact that the DA molar concentration increased after partial hydrolysis of DA-PLMA under the same mass concentration. As shown in Figure 5B (inset), the rosin-based ATRP initiator 2-BriBHDA (10 mg/ml) displayed the fluorescence in THF when excited at 365 nm, so was DA-PLMA-PMAA (1 mg/ml). Therefore, the fluorescent emission spectra of 2-BriBHDA, DA-PLMA (4 mg/ml), and DA-PLMA-PMAA (1 mg/ml) in THF (λ_ex_ = 365 nm) were recorded to verify the role of rosin in regard to the fluorescence property (Figure 5B). 2-BriBHDA has a λ_em_ of 405–430 nm in its fluorescent emission spectrum. As expected, the peaks around 405–430 nm were also presented in fluorescence spectra of DA-PLMA and DA-PLMA-PMAA, indicating that the fluorescence property was ascribed to the existence of rosin. The peak intensity of 405–430 nm was decreased for DA-PLMA but increased significantly for DA-PLMA-PMAA, which was consistent with that of the UV/vis analysis. Overall, the results from both the UV/vis and fluorescent analysis confirm the existence of DA before and after acid hydrolysis, and DA-PLMA-PMAA showed the self-fluorescence property which has promising application in the drug delivery field. It was normal that the Mn of DA-PLMA-PMAA (18,000 mol/g) was lower than that of DA-PLMA (25,000 mol/g) after hydrolysis. However, after hydrolysis, the PDI of DA-PLMA-PMAA (after hydrolysis) was still as low as 1.17 (Figure 5B), which meant the partial hydrolysis process was reliable for the following preparation of micelles.

### DA-PLMA-PMAA Micelles With pH Sensitivity and Self-Fluorescence

**FIGURE 6:**
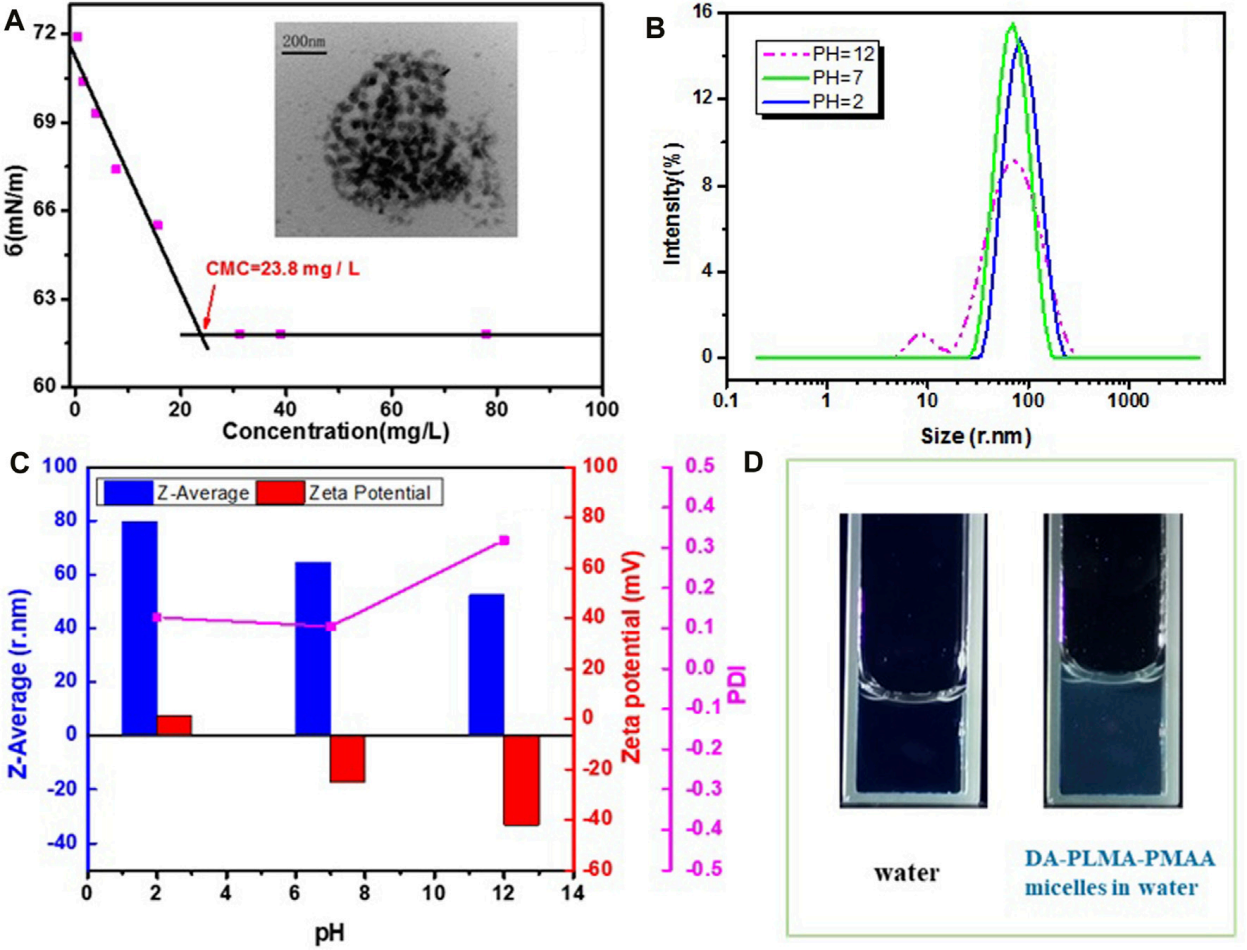
**(A)** TEM image of micelles (inset image) and surface tension vs. micelle concentration at pH = 7; **(B)** size distribution profiles by intensity of micelles in different pHs; **(C)** Z-average, PDI, and zeta potential of micelles in different pHs; and **(D)** fluorescence spectra of water and DA-PLMA-PMAA micelles in water (digital photograph taken under 365 nm).

After acid hydrolysis of DA-PLMA copolymer, the resulting products contain both hydrophilic poly methacrylic acid and hydrophobic poly lauryl methacrylate which were commonly known to self-assemble to form micelle structures in aqueous solution. Indeed, the THF solution of DA-PLMA-PMAA was added into deionized water at room temperature, and the mixture was stirred for 24 h for the evaporation of THF. The morphology of micelles was observed using transmission electron microscopy (TEM). Figure 6A (inset) showed the TEM image of DA-PLMA-PMAA in deionized water (pH = 7) with concentration of 0.1 mg/ml, from which the spherical micelles with the diameter of ∼40 nm were clearly seen. The hydrodynamic diameter (Z-average) of micelles obtained from DLS was ∼65 nm, and the PDI of micelles was as low as 0.105, which was much lower than that of sustainable micelles derived from chitosan or lignin (Zhou et al., 2019; Pornpitchanarong et al., 2020) and polymeric micelles prepared by Reversible Addition–Fragmentation Transfer (RAFT) polymerization (Wang C. et al., 2020). This probably ascribes to the relatively low PDI (1.17) of the DA-PLMA-PMAA copolymer. In addition, the zeta potential was −17.3 mV, and the negative surface could facilitate drug loading and the delivery process ( Yang et al., 2017).

Surface tension measurements were adopted to determine the critical micellar concentration, which is an important parameter to characterize the stability of polymeric micelles (Patist et al., 2000; Calvo et al., 2009). As shown in Figure 6A, the surface tension came to be a constant when the surface is saturated at a surface tension value of ∼61.8 mN/m in pH = 7. The critical micellar concentration (CMC) read from the point of intersection between the two linear lines in Figure 6A was 22.5 mg/L, which was in appropriate concentration, allowing their use in body fluids (Wei et al., 2007). In addition, the CMC of DA-PLMA-PMAA micelles was much lower than that of common surfactant sodium lauryl sulfate (CMC was 2.5 g/L), suggesting that DA-PLMA-PMAA micelles could be stable enough to serve as a drug carrier for drug delivery.

pH sensitivity is approved to be one of the most efficient stimuli for drug delivery. In order to investigate the pH influence on the formation of DA-PLMA-PMAA micelles, the size, size distribution, and zeta potential of the micelles in different pH were analyzed by DLS. As shown in Figures 6B,C, the hydrodynamic diameter (Z-average) was decreased with the increase of pH; inversely, PDI increased with the increase of pHs. Particularly, in acidic solutions (pH = 2 and 5), the Z-average value was ∼80 nm, while in basic solutions (pH = 10 and 12), the Z-average value decreased to ∼50 nm. This is mainly caused by conformational change of PMAA parts in acidic and basic solutions. It is well known that PMAA chains could be protonated and presenting expanding conformation in the acidic condition; however, in the basic condition, PMAA chains adopt a compact conformation due to the deprotonation of PMAA parts ( Xiong et al., 2011; Yuan et al., 2012; Nan et al., 2014). Besides, due to the protonation of PMAA parts at low pH, there were many positive charges in solution, resulting in a positive zeta potential value (7.53 mV, pH = 2; 0.25 mV, pH = 5). With increasing pH, carboxylic groups (–COOH) in PAA could be highly ionized, resulting in a negative zeta potential value of the micelles solution (−33.2 mV, pH = 10; −33.1 mV, pH = 12). The change of the Z-average value and the zeta potential value of micelles solution vs. pH confirmed the pH-dependent properties of micelles. As described above, DA-PLMA-PMAA showed an obvious self-fluorescence property under the excitation of 365 nm. The fluorescence property of DA-PLMA-PMAA micelles in water was also investigated, and the bright fluorescence was observed under a UV lamp (365 nm) (Figure 6D). It is well known that fluorescent property is highly desirable for both *in vitro* and *in vivo* applications of drug-loading micelles because it allows for easy tracking of the nanocarriers using microscopy (Chen et al., 2015). Therefore, the resulted micelles with good fluorescence property would find promising application in the field of drug delivery.

### DOX Loading and *In Vitro* Release Studies of DA-PLMA-PMAA Micelles

**FIGURE 7:**
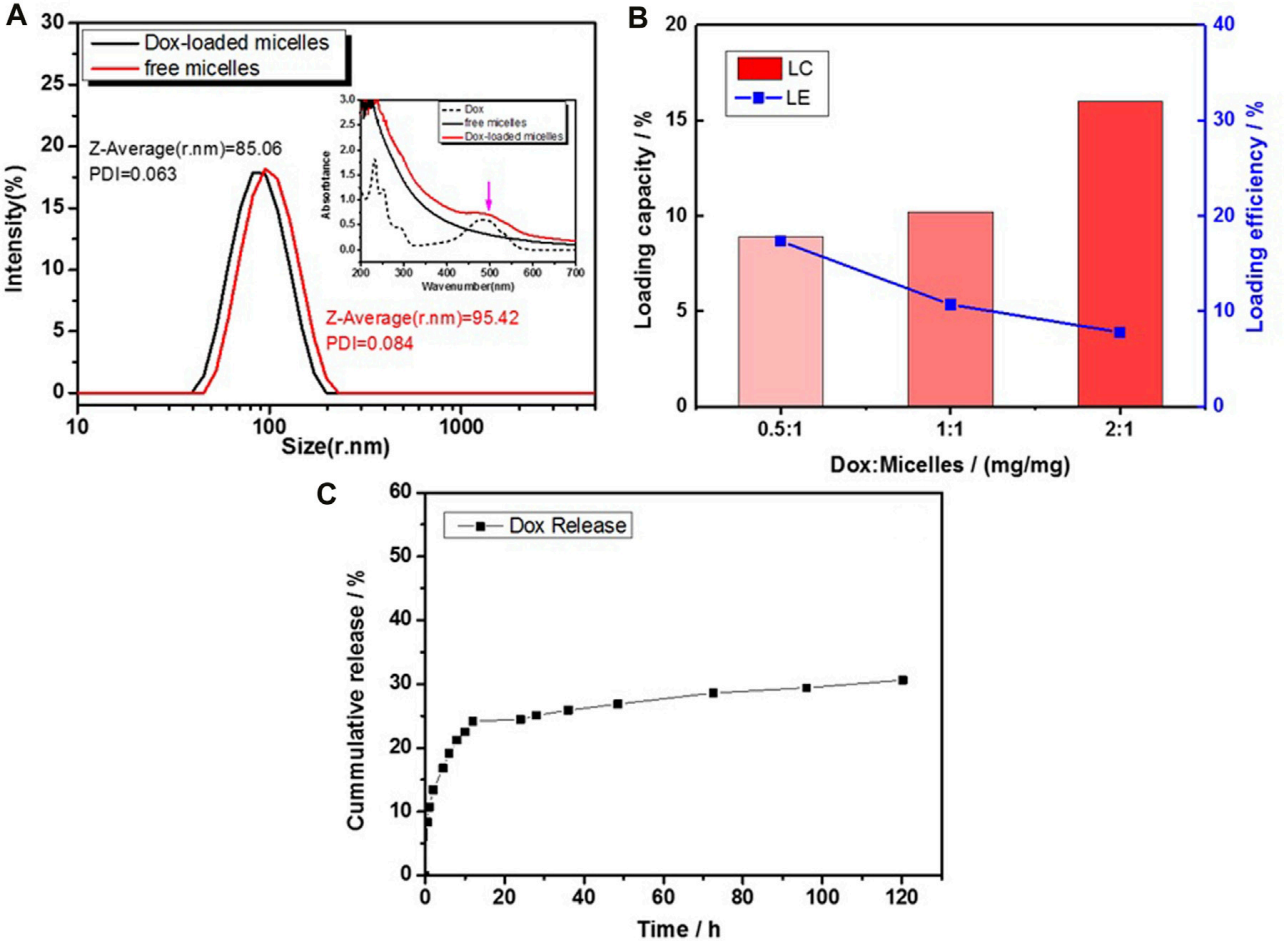
**(A)** Size distribution by intensity (inset: UV-vis spectrum) for free micelles and Dox-loaded micelles; **(B)** drug-loading capacity and efficiency of Dox-loaded micelles; and **(C)** release profile of Dox-loaded micelles in L in PBS (0.1 M, pH 7.4).

It is well known that micelles with large size could facilitate the drug release rate; however, particles with size over 200 nm are easily cleared by the reticuloendothelial system (RES) *in vivo* (Maeda and Matsumura, 2011). Therefore, the size of DA-PLMA-PMAA was optimized to be the Z-average value of ∼85 nm for the drug-loading experiment. Thereafter, hydrophobic doxorubicin (Dox), a popular model anticancer drug, was chosen as a model drug for fundamental studies of drug loading and releasing properties of DA-PLMA-PMAA micelles. The size and PDI for Dox-loaded micelles were increased to ∼95 nm and 0.084 (Figure 7A), indicating that Dox was successfully loaded into micelles. The UV-vis spectrum of Dox, free micelles, and Dox-loaded micelles in Figure 7A (inset) showed the presence of Dox characteristic absorption peak at ∼495 nm for Dox and Dox-loaded micelles samples. This further confirmed a successful entrapment of Dox into the micelles.

The drug-loading capacity and drug-loading efficiency of DOX were analyzed by the UV-vis spectrophotometer at 495 nm, and the results are summarized in Figure 7B. The drug-loading capacity (LC) of DOX increased from 8.9 wt% to 16.0 wt%, corresponding to different feeding Dox concentrations at pH = 7.0, while the drug-loading efficiencies (LEs) decreased from 17.35 to 7.80%. Commonly, physical entrapment of drugs in polymeric micelles is driven by the hydrophobic interactions as well as the electrostatic interaction between drugs and the polymer micelles. The LC of DA-PLMA-PMAA micelles was superior to that of most reported Dox-loaded polymeric micelles prepared by ATRP or other polymerization methods (Table 1). It is probably the similarity of aromatic chemical structures between DA and Dox that contributes to the good LC property of these micelles. As reported by Cuong et al. (2012), the aromatic folic acid could improve the LC of PEG–PCL polymeric nanocarrier from 5.5 to 10.9%. Overall, the results indicated the feasibility of DA-PLMA-PMAA micelles as drug carriers.

**TABLE 1:** Drug-loading capacity (LC) of reported polymeric micelles.

Entry	Polymer micelles	Polymerization method	Functional	LC (wt%)	Reference
1	PEG-b-PEYM	ATRP	pH-sensitive	2.6	Tang et al. (2011)
2	POEOMA nano-hydrogels	ATRP	—	5.4–16.4	Oh et al. (2007)
3	mPEG-PCL-PDMA/mPEG-PCL-PVBA-Dox	RAFT	pH-sensitive	8.1–10.1	Wang et al. (2020a)
4	Folate-decorated star-shaped PEG–PCL	Free-radical polymerization	—	4.6–13.0	Cuong et al. (2012)
5	P(MPC-co-PCL)	Free-radical polymerization	—	6.7–12.6	Zhao et al. (2016)
6	mPEG-b-P(HPMA-g-a-TOS-g-His)	Free-radical polymerization	pH-sensitive	9.6	Debele et al. (2017)
7	mPEG-PAsp(MEA) -PAsp(DIP)	Ring-opening polymerization	Reduction and pH sensitivity	10.5	Dai et al. (2011)
8	H40-BPLP-PEG-OCH_3_/cRGD	Commercial PEG	Self-fluorescent and pH-sensitive	15.7	Chen et al. (2015)
9	LHRH-PEG-PHIS-Dox	Commercial PEG	pH-sensitive	28	Yang et al. (2015)
10	DA-PLMA-PMAA	ATRP	Self-fluorescent and pH-sensitive	8.9–16.0	This study

To mimic the physiological blood environment, the *in vitro* release profiles of Dox-loaded polymeric micelles were examined *in vitro* at 37°C *via* a dialysis method using PBS buffers at pH 7.4 (Figure 7C). The results showed an initial burst release of Dox (within 12 h), followed by a sustained and slow release over a prolonged time (until 120 h). Around 24% of Dox was released within first 12 h, which could be attributed to the diffusion of Dox located close to the surface of the micelles. However, the amount of Dox released after 120 h was only 30.6%, indicating that DA-PLMA-PMAA micelles were relatively stable under physiological conditions. This can reduce the loss of drug during circulation in the blood stream (pH 7.4), which was highly desirable for drug carriers.

## Summary

In summary, a facile strategy of preparation multifunctional polymeric micelles was successfully demonstrated. Sustainable dehydroabietic acid–based poly lauryl methacrylate (DA-PLMA) with narrow PDI of 1.13 was prepared first *via* ATRP of hydrophobic lauryl methacrylate using rosin as an initiator. After simple acid hydrolysis, hydrophilic poly methacrylic acid (PMAA) moieties were formed, resulting in an amphiphilic polymer (DA-PLMA-PMAA) which spontaneously self-assembled into pH-sensitive micelles in aqueous solutions. The spherical micelles had an average diameter of ∼65 nm and PDI as low as 0.105, and showed self-fluorescence properties since they contained rosin segments. The Dox-loading capacity was as high as 16.0 wt%, indicating that DA-PLMA-PMAA had a good drug-loading ability. The *in vitro* drug release study revealed that DA-PLMA-PMAA micelles were relatively stable under physiological conditions showing sustained-release characteristic. This study not only supported the DA-PLMA-PMAA micelles as a promising efficient drug delivery carrier but also provided a facile approach to utilize renewable rosin and vegetable oils.

## Data Availability

The original contributions presented in the study are included in the article/Supplementary Material; further inquiries can be directed to the corresponding authors.
