# Supplementary material for: Synthesis of pH-Sensitive and Self-Fluorescent Polymeric Micelles Derived From Rosin and Vegetable Oils via ATRP
Source: Front Bioeng Biotechnol. 2021 Nov 2;9:753808. doi: 10.3389/fbioe.2021.753808 (PMC8596564; doi:10.3389/fbioe.2021.753808)
Supplement: Supplementary file 2 [file DataSheet1.ZIP › ╘¡╩╝╩2╛▌/Table.docx]

| **Entry** | **Polymer micelles** | **Polymerization**  **method** | **Functional** | **LC (wt%)** | **Reference** |
| --- | --- | --- | --- | --- | --- |
| 1 | PEG-b-PEYM | ATRP | pH-sensitive | 2.6 | ([Tang et al., 2011](#_ENREF_20)) |
| 2 | POEOMA nano-hydrogels | ATRP | -- | 5.4~16.4 | ([Oh et al., 2007](#_ENREF_15)) |
| 3 | mPEG-PCL-PDMA/  mPEG-PCL-PVBA-Dox | RAFT | pH-sensitive | 8.1-10.1 | ([Wang et al., 2020a](#_ENREF_23)) |
| 4 | folate-decorated  star-shaped PEG–PCL | free radical polymerization | -- | 4.6~13.0 | ([Cuong et al., 2012](#_ENREF_6)) |
| 5 | P(MPC-co-PCL) | free radical polymerization | -- | 6.7~12.6 | ([Zhao et al., 2016](#_ENREF_33)) |
| 6 | mPEG-b-P(HPMA-g-  a-TOS-g-His) | free radical polymerization | pH-sensitive | 9.6 | ([Debele et al., 2017](#_ENREF_8)) |
| 7 | mPEG-PAsp(MEA)-  PAsp(DIP) | ring-opening polymerization | reduction and pH sensitivity | 10.5 | ([Dai et al., 2011](#_ENREF_7)) |
| 8 | H40-BPLP-PEG-OCH_3_  /cRGD | commercial PEG | self-fluorescent  pH-sensitive | 15.7 | ([Chen et al., 2015](#_ENREF_5)) |
| 9 | LHRH-PEG-PHIS-Dox | commercial PEG | pH-sensitive | 28 | ([Yang et al., 2015](#_ENREF_29)) |
| 10 | DA-PLMA-PMAA | ATRP | self-fluorescent  pH-sensitive | 8.9-16.0 | This work |

**Table 1.** Drug loading capacity (LC) of reported polymeric micelles
